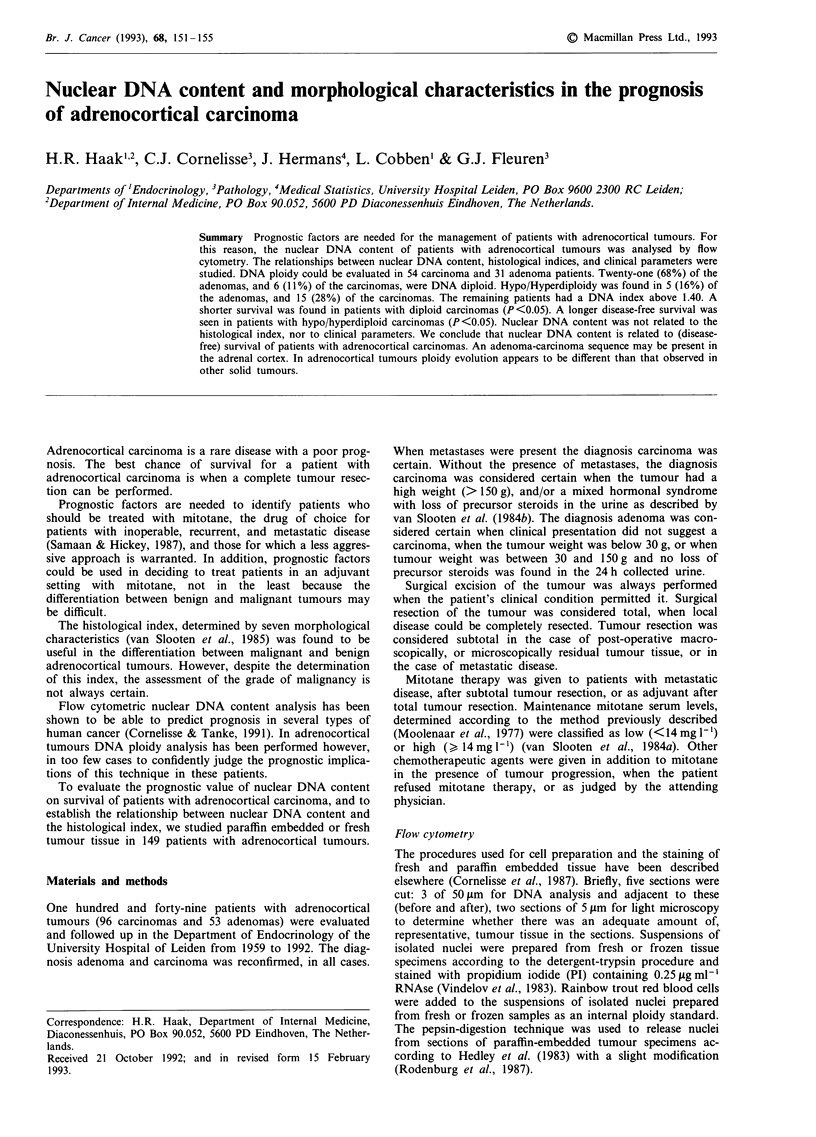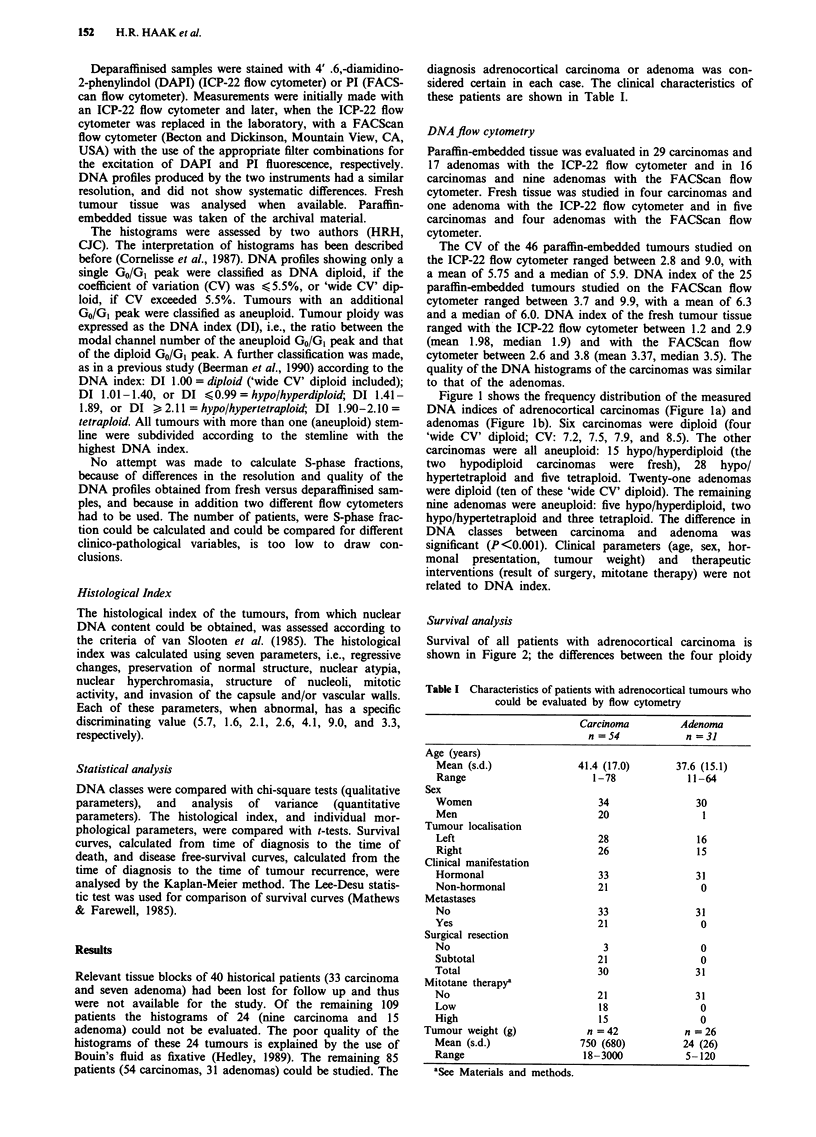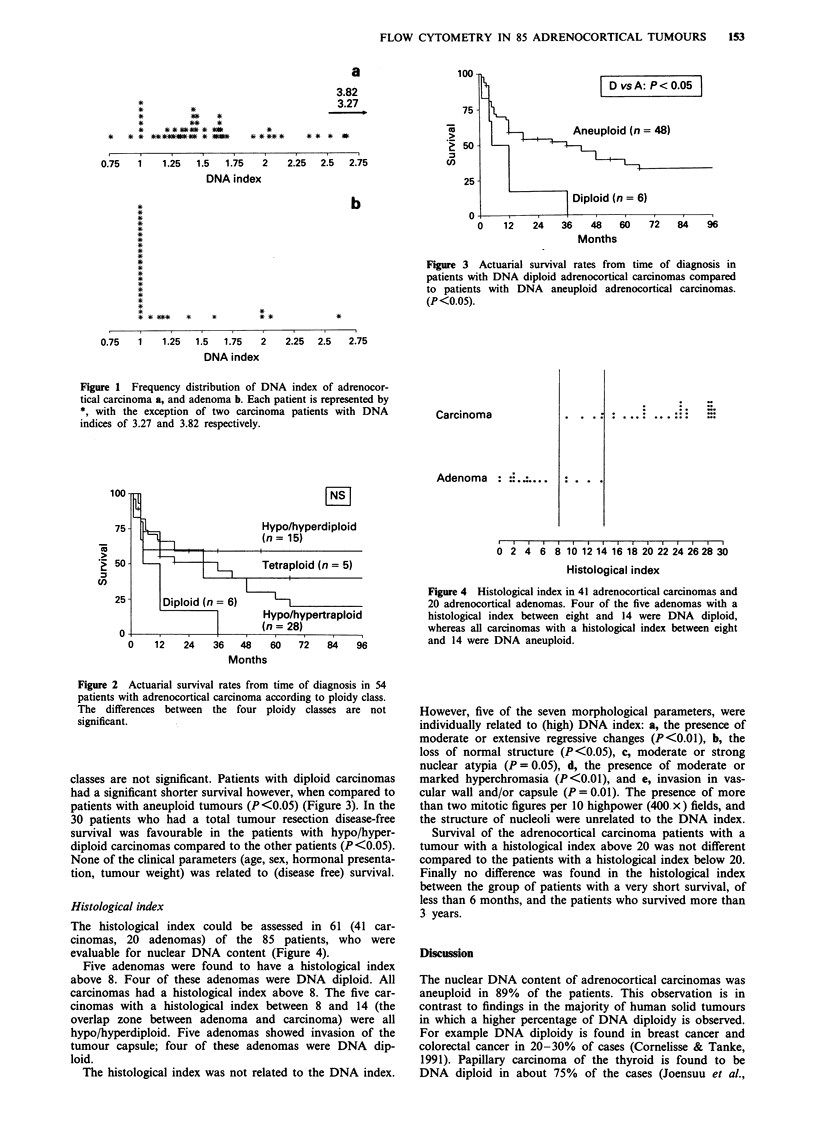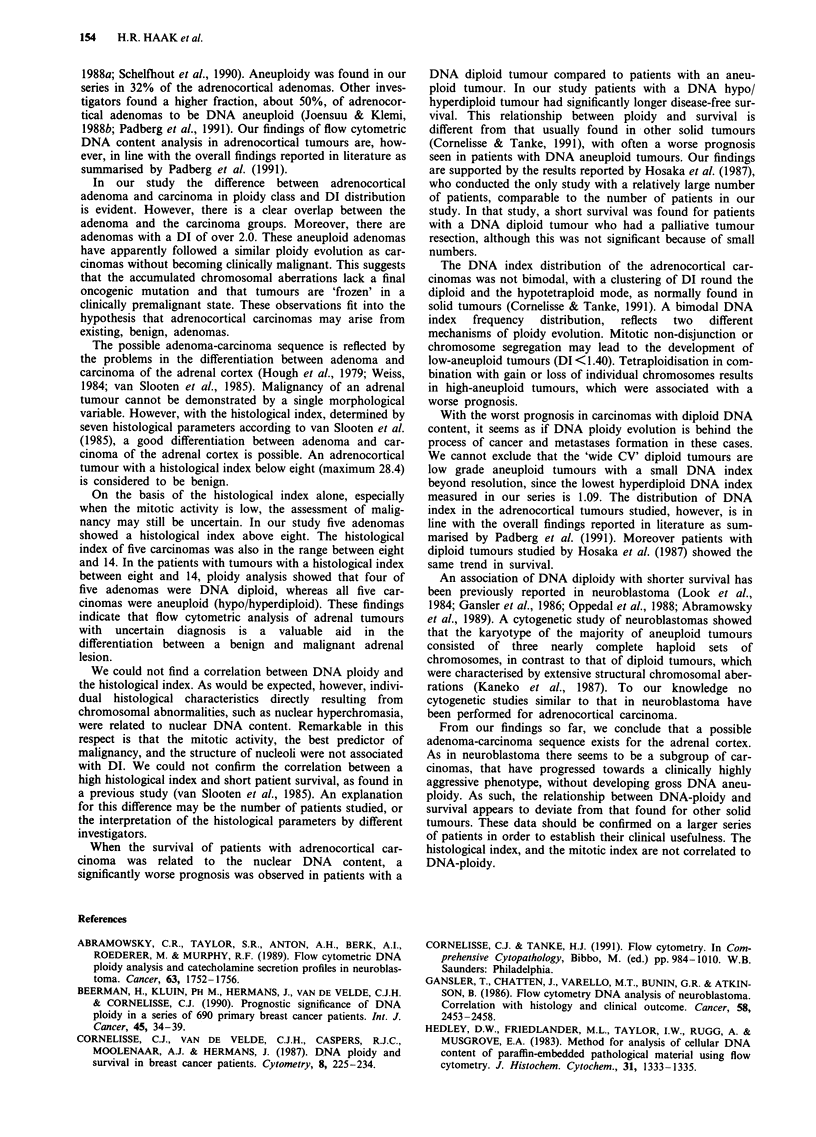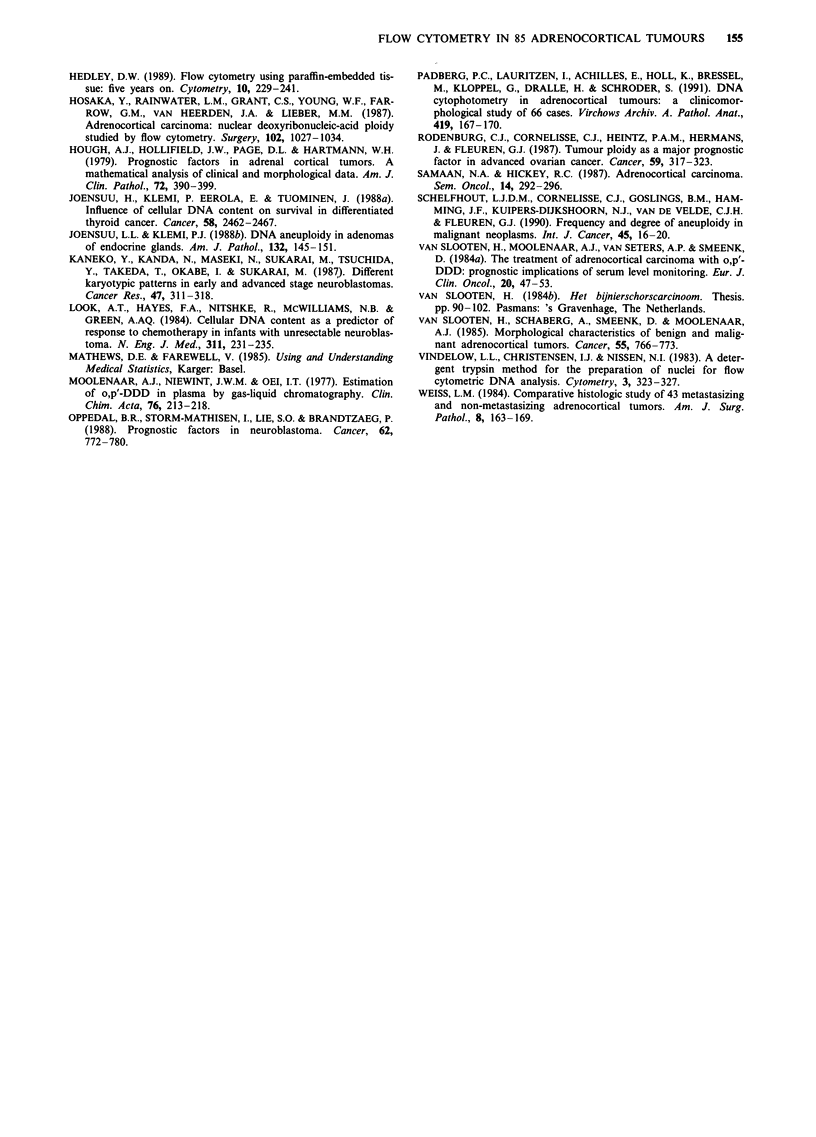# Nuclear DNA content and morphological characteristics in the prognosis of adrenocortical carcinoma.

**DOI:** 10.1038/bjc.1993.304

**Published:** 1993-07

**Authors:** H. R. Haak, C. J. Cornelisse, J. Hermans, L. Cobben, G. J. Fleuren

**Affiliations:** Department of Endocrinology, University Hospital Leiden, The Netherlands.

## Abstract

Prognostic factors are needed for the management of patients with adrenocortical tumours. For this reason, the nuclear DNA content of patients with adrenocortical tumours was analysed by flow cytometry. The relationships between nuclear DNA content, histological indices, and clinical parameters were studied. DNA ploidy could be evaluated in 54 carcinoma and 31 adenoma patients. Twenty-one (68%) of the adenomas, and 6 (11%) of the carcinomas, were DNA diploid. Hypo/Hyperdiploidy was found in 5 (16%) of the adenomas, and 15 (28%) of the carcinomas. The remaining patients had a DNA index above 1.40. A shorter survival was found in patients with diploid carcinomas (P < 0.05). A longer disease-free survival was seen in patients with hypo/hyperdiploid carcinomas (P < 0.05). Nuclear DNA content was not related to the histological index, nor to clinical parameters. We conclude that nuclear DNA content is related to (disease-free) survival of patients with adrenocortical carcinomas. An adenoma-carcinoma sequence may be present in the adrenal cortex. In adrenocortical tumours ploidy evolution appears to be different than that observed in other solid tumours.


Br. J. Cancer (1993), 68, 151–155 © Macmillan Press Ltd., 1993

### Nuclear DNA content and morphological characteristics in the prognosis of adrenocortical carcinoma

H.R. Haak[1,2], C.J. Cornelisse[3], J. Hermans[4], L. Cobben[1] & G.J. Fleuren[3]

*Departments of [1]Endocrinology, [3]Pathology, [4]Medical Statistics, University Hospital Leiden, PO Box 9600 2300 RC Leiden; [2]Department of Internal Medicine, PO Box 90.052, 5600 PD Diaconessenhuis Eindhoven, The Netherlands.*

**Summary** Prognostic factors are needed for the management of patients with adrenocortical tumours. For this reason, the nuclear DNA content of patients with adrenocortical tumours was analysed by flow cytometry. The relationships between nuclear DNA content, histological indices, and clinical parameters were studied. DNA ploidy could be evaluated in 54 carcinoma and 31 adenoma patients. Twenty-one (68%) of the adenomas, and 6 (11%) of the carcinomas, were DNA diploid. Hypo/Hyperdiploidy was found in 5 (16%) of the adenomas, and 15 (28%) of the carcinomas. The remaining patients had a DNA index above 1.40. A shorter survival was found in patients with diploid carcinomas ($P<0.05$). A longer disease-free survival was seen in patients with hypo/hyperdiploid carcinomas ($P<0.05$). Nuclear DNA content was not related to the histological index, nor to clinical parameters. We conclude that nuclear DNA content is related to (disease-free) survival of patients with adrenocortical carcinomas. An adenoma-carcinoma sequence may be present in the adrenal cortex. In adrenocortical tumours ploidy evolution appears to be different than that observed in other solid tumours.

Adrenocortical carcinoma is a rare disease with a poor prognosis. The best chance of survival for a patient with adrenocortical carcinoma is when a complete tumour resection can be performed.

Prognostic factors are needed to identify patients who should be treated with mitotane, the drug of choice for patients with inoperable, recurrent, and metastatic disease (Samaan & Hickey, 1987), and those for which a less aggressive approach is warranted. In addition, prognostic factors could be used in deciding to treat patients in an adjuvant setting with mitotane, not in the least because the differentiation between benign and malignant tumours may be difficult.

The histological index, determined by seven morphological characteristics (van Slooten *et al.*, 1985) was found to be useful in the differentiation between malignant and benign adrenocortical tumours. However, despite the determination of this index, the assessment of the grade of malignancy is not always certain.

Flow cytometric nuclear DNA content analysis has been shown to be able to predict prognosis in several types of human cancer (Cornelisse & Tanke, 1991). In adrenocortical tumours DNA ploidy analysis has been performed however, in too few cases to confidently judge the prognostic implications of this technique in these patients.

To evaluate the prognostic value of nuclear DNA content on survival of patients with adrenocortical carcinoma, and to establish the relationship between nuclear DNA content and the histological index, we studied paraffin embedded or fresh tumour tissue in 149 patients with adrenocortical tumours.

### Materials and methods

One hundred and forty-nine patients with adrenocortical tumours (96 carcinomas and 53 adenomas) were evaluated and followed up in the Department of Endocrinology of the University Hospital of Leiden from 1959 to 1992. The diagnosis adenoma and carcinoma was reconfirmed, in all cases. When metastases were present the diagnosis carcinoma was certain. Without the presence of metastases, the diagnosis carcinoma was considered certain when the tumour had a high weight (>150 g), and/or a mixed hormonal syndrome with loss of precursor steroids in the urine as described by van Slooten *et al.* (1984*b*). The diagnosis adenoma was considered certain when clinical presentation did not suggest a carcinoma, when the tumour weight was below 30 g, or when tumour weight was between 30 and 150 g and no loss of precursor steroids was found in the 24 h collected urine.

Surgical excision of the tumour was always performed when the patient's clinical condition permitted it. Surgical resection of the tumour was considered total, when local disease could be completely resected. Tumour resection was considered subtotal in the case of post-operative macroscopically, or microscopically residual tumour tissue, or in the case of metastatic disease.

Mitotane therapy was given to patients with metastatic disease, after subtotal tumour resection, or as adjuvant after total tumour resection. Maintenance mitotane serum levels, determined according to the method previously described (Moolenaar *et al.*, 1977) were classified as low ($<14\,mg\,l^{-1}$) or high ($\geq 14\,mg\,l^{-1}$) (van Slooten *et al.*, 1984*a*). Other chemotherapeutic agents were given in addition to mitotane in the presence of tumour progression, when the patient refused mitotane therapy, or as judged by the attending physician.

### *Flow cytometry*

The procedures used for cell preparation and the staining of fresh and paraffin embedded tissue have been described elsewhere (Cornelisse *et al.*, 1987). Briefly, five sections were cut: 3 of 50 μm for DNA analysis and adjacent to these (before and after), two sections of 5 μm for light microscopy to determine whether there was an adequate amount of, representative, tumour tissue in the sections. Suspensions of isolated nuclei were prepared from fresh or frozen tissue specimens according to the detergent-trypsin procedure and stained with propidium iodide (PI) containing $0.25\,\mu g\,ml^{-1}$ RNAse (Vindelov *et al.*, 1983). Rainbow trout red blood cells were added to the suspensions of isolated nuclei prepared from fresh or frozen samples as an internal ploidy standard. The pepsin-digestion technique was used to release nuclei from sections of paraffin-embedded tumour specimens according to Hedley *et al.* (1983) with a slight modification (Rodenburg *et al.*, 1987).

Correspondence: H.R. Haak, Department of Internal Medicine, Diaconessenhuis, PO Box 90.052, 5600 PD Eindhoven, The Netherlands.
Received 21 October 1992; and in revised form 15 February 1993.

Deparaffinised samples were stained with 4′ .6,-diamidino-2-phenylindol (DAPI) (ICP-22 flow cytometer) or PI (FACScan flow cytometer). Measurements were initially made with an ICP-22 flow cytometer and later, when the ICP-22 flow cytometer was replaced in the laboratory, with a FACScan flow cytometer (Becton and Dickinson, Mountain View, CA, USA) with the use of the appropriate filter combinations for the excitation of DAPI and PI fluorescence, respectively. DNA profiles produced by the two instruments had a similar resolution, and did not show systematic differences. Fresh tumour tissue was analysed when available. Paraffin-embedded tissue was taken of the archival material.

The histograms were assessed by two authors (HRH, CJC). The interpretation of histograms has been described before (Cornelisse *et al.*, 1987). DNA profiles showing only a single $G_0/G_1$ peak were classified as DNA diploid, if the coefficient of variation (CV) was $\leqslant$5.5%, or 'wide CV' diploid, if CV exceeded 5.5%. Tumours with an additional $G_0/G_1$ peak were classified as aneuploid. Tumour ploidy was expressed as the DNA index (DI), i.e., the ratio between the modal channel number of the aneuploid $G_0/G_1$ peak and that of the diploid $G_0/G_1$ peak. A further classification was made, as in a previous study (Beerman *et al.*, 1990) according to the DNA index: DI 1.00 = *diploid* ('wide CV' diploid included); DI 1.01–1.40, or DI $\leqslant$0.99 = *hypo/hyperdiploid*; DI 1.41–1.89, or DI $\geqslant$2.11 = *hypo/hypertetraploid*; DI 1.90–2.10 = *tetraploid*. All tumours with more than one (aneuploid) stemline were subdivided according to the stemline with the highest DNA index.

No attempt was made to calculate S-phase fractions, because of differences in the resolution and quality of the DNA profiles obtained from fresh versus deparaffinised samples, and because in addition two different flow cytometers had to be used. The number of patients, were S-phase fraction could be calculated and could be compared for different clinico-pathological variables, is too low to draw conclusions.

### *Histological Index*

The histological index of the tumours, from which nuclear DNA content could be obtained, was assessed according to the criteria of van Slooten *et al.* (1985). The histological index was calculated using seven parameters, i.e., regressive changes, preservation of normal structure, nuclear atypia, nuclear hyperchromasia, structure of nucleoli, mitotic activity, and invasion of the capsule and/or vascular walls. Each of these parameters, when abnormal, has a specific discriminating value (5.7, 1.6, 2.1, 2.6, 4.1, 9.0, and 3.3, respectively).

### *Statistical analysis*

DNA classes were compared with chi-square tests (qualitative parameters), and analysis of variance (quantitative parameters). The histological index, and individual morphological parameters, were compared with *t*-tests. Survival curves, calculated from time of diagnosis to the time of death, and disease free-survival curves, calculated from the time of diagnosis to the time of tumour recurrence, were analysed by the Kaplan-Meier method. The Lee-Desu statistic test was used for comparison of survival curves (Mathews & Farewell, 1985).

### Results

Relevant tissue blocks of 40 historical patients (33 carcinoma and seven adenoma) had been lost for follow up and thus were not available for the study. Of the remaining 109 patients the histograms of 24 (nine carcinoma and 15 adenoma) could not be evaluated. The poor quality of the histograms of these 24 tumours is explained by the use of Bouin's fluid as fixative (Hedley, 1989). The remaining 85 patients (54 carcinomas, 31 adenomas) could be studied. The diagnosis adrenocortical carcinoma or adenoma was considered certain in each case. The clinical characteristics of these patients are shown in Table I.

### *DNA flow cytometry*

Paraffin-embedded tissue was evaluated in 29 carcinomas and 17 adenomas with the ICP-22 flow cytometer and in 16 carcinomas and nine adenomas with the FACScan flow cytometer. Fresh tissue was studied in four carcinomas and one adenoma with the ICP-22 flow cytometer and in five carcinomas and four adenomas with the FACScan flow cytometer.

The CV of the 46 paraffin-embedded tumours studied on the ICP-22 flow cytometer ranged between 2.8 and 9.0, with a mean of 5.75 and a median of 5.9. DNA index of the 25 paraffin-embedded tumours studied on the FACScan flow cytometer ranged between 3.7 and 9.9, with a mean of 6.3 and a median of 6.0. DNA index of the fresh tumour tissue ranged with the ICP-22 flow cytometer between 1.2 and 2.9 (mean 1.98, median 1.9) and with the FACScan flow cytometer between 2.6 and 3.8 (mean 3.37, median 3.5). The quality of the DNA histograms of the carcinomas was similar to that of the adenomas.

Figure 1 shows the frequency distribution of the measured DNA indices of adrenocortical carcinomas (Figure 1a) and adenomas (Figure 1b). Six carcinomas were diploid (four 'wide CV' diploid; CV: 7.2, 7.5, 7.9, and 8.5). The other carcinomas were all aneuploid: 15 hypo/hyperdiploid (the two hypodiploid carcinomas were fresh), 28 hypo/hypertetraploid and five tetraploid. Twenty-one adenomas were diploid (ten of these 'wide CV' diploid). The remaining nine adenomas were aneuploid: five hypo/hyperdiploid, two hypo/hypertetraploid and three tetraploid. The difference in DNA classes between carcinoma and adenoma was significant ($P<0.001$). Clinical parameters (age, sex, hormonal presentation, tumour weight) and therapeutic interventions (result of surgery, mitotane therapy) were not related to DNA index.

### *Survival analysis*

Survival of all patients with adrenocortical carcinoma is shown in Figure 2; the differences between the four ploidy

**Table I** Characteristics of patients with adrenocortical tumours who could be evaluated by flow cytometry

| | *Carcinoma* *n = 54* | *Adenoma* *n = 31* |
|---|---|---|
| Age (years) | | |
| Mean (s.d.) | 41.4 (17.0) | 37.6 (15.1) |
| Range | 1–78 | 11–64 |
| Sex | | |
| Women | 34 | 30 |
| Men | 20 | 1 |
| Tumour localisation | | |
| Left | 28 | 16 |
| Right | 26 | 15 |
| Clinical manifestation | | |
| Hormonal | 33 | 31 |
| Non-hormonal | 21 | 0 |
| Metastases | | |
| No | 33 | 31 |
| Yes | 21 | 0 |
| Surgical resection | | |
| No | 3 | 0 |
| Subtotal | 21 | 0 |
| Total | 30 | 31 |
| Mitotane therapy[a] | | |
| No | 21 | 31 |
| Low | 18 | 0 |
| High | 15 | 0 |
| Tumour weight (g) | *n* = 42 | *n* = 26 |
| Mean (s.d.) | 750 (680) | 24 (26) |
| Range | 18–3000 | 5–120 |

[a]See Materials and methods.

**Figure 1** Frequency distribution of DNA index of adrenocortical carcinoma **a**, and adenoma **b**. Each patient is represented by *, with the exception of two carcinoma patients with DNA indices of 3.27 and 3.82 respectively.

**Figure 2** Actuarial survival rates from time of diagnosis in 54 patients with adrenocortical carcinoma according to ploidy class. The differences between the four ploidy classes are not significant.

**Figure 3** Actuarial survival rates from time of diagnosis in patients with DNA diploid adrenocortical carcinomas compared to patients with DNA aneuploid adrenocortical carcinomas. ($P<0.05$).

**Figure 4** Histological index in 41 adrenocortical carcinomas and 20 adrenocortical adenomas. Four of the five adenomas with a histological index between eight and 14 were DNA diploid, whereas all carcinomas with a histological index between eight and 14 were DNA aneuploid.

classes are not significant. Patients with diploid carcinomas had a significant shorter survival however, when compared to patients with aneuploid tumours ($P<0.05$) (Figure 3). In the 30 patients who had a total tumour resection disease-free survival was favourable in the patients with hypo/hyperdiploid carcinomas compared to the other patients ($P<0.05$). None of the clinical parameters (age, sex, hormonal presentation, tumour weight) was related to (disease free) survival.

### *Histological index*

The histological index could be assessed in 61 (41 carcinomas, 20 adenomas) of the 85 patients, who were evaluable for nuclear DNA content (Figure 4).

Five adenomas were found to have a histological index above 8. Four of these adenomas were DNA diploid. All carcinomas had a histological index above 8. The five carcinomas with a histological index between 8 and 14 (the overlap zone between adenoma and carcinoma) were all hypo/hyperdiploid. Five adenomas showed invasion of the tumour capsule; four of these adenomas were DNA diploid.

The histological index was not related to the DNA index. However, five of the seven morphological parameters, were individually related to (high) DNA index: **a**, the presence of moderate or extensive regressive changes ($P<0.01$), **b**, the loss of normal structure ($P<0.05$), **c**, moderate or strong nuclear atypia ($P=0.05$), **d**, the presence of moderate or marked hyperchromasia ($P<0.01$), and **e**, invasion in vascular wall and/or capsule ($P=0.01$). The presence of more than two mitotic figures per 10 highpower (400 ×) fields, and the structure of nucleoli were unrelated to the DNA index.

Survival of the adrenocortical carcinoma patients with a tumour with a histological index above 20 was not different compared to the patients with a histological index below 20. Finally no difference was found in the histological index between the group of patients with a very short survival, of less than 6 months, and the patients who survived more than 3 years.

### Discussion

The nuclear DNA content of adrenocortical carcinomas was aneuploid in 89% of the patients. This observation is in contrast to findings in the majority of human solid tumours in which a higher percentage of DNA diploidy is observed. For example DNA diploidy is found in breast cancer and colorectal cancer in 20–30% of cases (Cornelisse & Tanke, 1991). Papillary carcinoma of the thyroid is found to be DNA diploid in about 75% of the cases (Joensuu *et al.*,

1988*a*; Schelfhout *et al.*, 1990). Aneuploidy was found in our series in 32% of the adrenocortical adenomas. Other investigators found a higher fraction, about 50%, of adrenocortical adenomas to be DNA aneuploid (Joensuu & Klemi, 1988*b*; Padberg *et al.*, 1991). Our findings of flow cytometric DNA content analysis in adrenocortical tumours are, however, in line with the overall findings reported in literature as summarised by Padberg *et al.* (1991).

In our study the difference between adrenocortical adenoma and carcinoma in ploidy class and DI distribution is evident. However, there is a clear overlap between the adenoma and the carcinoma groups. Moreover, there are adenomas with a DI of over 2.0. These aneuploid adenomas have apparently followed a similar ploidy evolution as carcinomas without becoming clinically malignant. This suggests that the accumulated chromosomal aberrations lack a final oncogenic mutation and that tumours are 'frozen' in a clinically premalignant state. These observations fit into the hypothesis that adrenocortical carcinomas may arise from existing, benign, adenomas.

The possible adenoma-carcinoma sequence is reflected by the problems in the differentiation between adenoma and carcinoma of the adrenal cortex (Hough *et al.*, 1979; Weiss, 1984; van Slooten *et al.*, 1985). Malignancy of an adrenal tumour cannot be demonstrated by a single morphological variable. However, with the histological index, determined by seven histological parameters according to van Slooten *et al.* (1985), a good differentiation between adenoma and carcinoma of the adrenal cortex is possible. An adrenocortical tumour with a histological index below eight (maximum 28.4) is considered to be benign.

On the basis of the histological index alone, especially when the mitotic activity is low, the assessment of malignancy may still be uncertain. In our study five adenomas showed a histological index above eight. The histological index of five carcinomas was also in the range between eight and 14. In the patients with tumours with a histological index between eight and 14, ploidy analysis showed that four of five adenomas were DNA diploid, whereas all five carcinomas were aneuploid (hypo/hyperdiploid). These findings indicate that flow cytometric analysis of adrenal tumours with uncertain diagnosis is a valuable aid in the differentiation between a benign and malignant adrenal lesion.

We could not find a correlation between DNA ploidy and the histological index. As would be expected, however, individual histological characteristics directly resulting from chromosomal abnormalities, such as nuclear hyperchromasia, were related to nuclear DNA content. Remarkable in this respect is that the mitotic activity, the best predictor of malignancy, and the structure of nucleoli were not associated with DI. We could not confirm the correlation between a high histological index and short patient survival, as found in a previous study (van Slooten *et al.*, 1985). An explanation for this difference may be the number of patients studied, or the interpretation of the histological parameters by different investigators.

When the survival of patients with adrenocortical carcinoma was related to the nuclear DNA content, a significantly worse prognosis was observed in patients with a DNA diploid tumour compared to patients with an aneuploid tumour. In our study patients with a DNA hypo/hyperdiploid tumour had significantly longer disease-free survival. This relationship between ploidy and survival is different from that usually found in other solid tumours (Cornelisse & Tanke, 1991), with often a worse prognosis seen in patients with DNA aneuploid tumours. Our findings are supported by the results reported by Hosaka *et al.* (1987), who conducted the only study with a relatively large number of patients, comparable to the number of patients in our study. In that study, a short survival was found for patients with a DNA diploid tumour who had a palliative tumour resection, although this was not significant because of small numbers.

The DNA index distribution of the adrenocortical carcinomas was not bimodal, with a clustering of DI round the diploid and the hypotetraploid mode, as normally found in solid tumours (Cornelisse & Tanke, 1991). A bimodal DNA index frequency distribution, reflects two different mechanisms of ploidy evolution. Mitotic non-disjunction or chromosome segregation may lead to the development of low-aneuploid tumours (DI $<$1.40). Tetraploidisation in combination with gain or loss of individual chromosomes results in high-aneuploid tumours, which were associated with a worse prognosis.

With the worst prognosis in carcinomas with diploid DNA content, it seems as if DNA ploidy evolution is behind the process of cancer and metastases formation in these cases. We cannot exclude that the 'wide CV' diploid tumours are low grade aneuploid tumours with a small DNA index beyond resolution, since the lowest hyperdiploid DNA index measured in our series is 1.09. The distribution of DNA index in the adrenocortical tumours studied, however, is in line with the overall findings reported in literature as summarised by Padberg *et al.* (1991). Moreover patients with diploid tumours studied by Hosaka *et al.* (1987) showed the same trend in survival.

An association of DNA diploidy with shorter survival has been previously reported in neuroblastoma (Look *et al.*, 1984; Gansler *et al.*, 1986; Oppedal *et al.*, 1988; Abramowsky *et al.*, 1989). A cytogenetic study of neuroblastomas showed that the karyotype of the majority of aneuploid tumours consisted of three nearly complete haploid sets of chromosomes, in contrast to that of diploid tumours, which were characterised by extensive structural chromosomal aberrations (Kaneko *et al.*, 1987). To our knowledge no cytogenetic studies similar to that in neuroblastoma have been performed for adrenocortical carcinoma.

From our findings so far, we conclude that a possible adenoma-carcinoma sequence exists for the adrenal cortex. As in neuroblastoma there seems to be a subgroup of carcinomas, that have progressed towards a clinically highly aggressive phenotype, without developing gross DNA aneuploidy. As such, the relationship between DNA-ploidy and survival appears to deviate from that found for other solid tumours. These data should be confirmed on a larger series of patients in order to establish their clinical usefulness. The histological index, and the mitotic index are not correlated to DNA-ploidy.

### References

ABRAMOWSKY, C.R., TAYLOR, S.R., ANTON, A.H., BERK, A.I., ROEDERER, M. & MURPHY, R.F. (1989). Flow cytometric DNA ploidy analysis and catecholamine secretion profiles in neuroblastoma. *Cancer*, **63**, 1752–1756.

BEERMAN, H., KLUIN, PH M., HERMANS, J., VAN DE VELDE, C.J.H. & CORNELISSE, C.J. (1990). Prognostic significance of DNA ploidy in a series of 690 primary breast cancer patients. *Int. J. Cancer*, **45**, 34–39.

CORNELISSE, C.J., VAN DE VELDE, C.J.H., CASPERS, R.J.C., MOOLENAAR, A.J. & HERMANS, J. (1987). DNA ploidy and survival in breast cancer patients. *Cytometry*, **8**, 225–234.

CORNELISSE, C.J. & TANKE, H.J. (1991). Flow cytometry. In *Comprehensive Cytopathology*, Bibbo, M. (ed.) pp. 984–1010. W.B. Saunders: Philadelphia.

GANSLER, T., CHATTEN, J., VARELLO, M.T., BUNIN, G.R. & ATKINSON, B. (1986). Flow cytometry DNA analysis of neuroblastoma. Correlation with histology and clinical outcome. *Cancer*, **58**, 2453–2458.

HEDLEY, D.W., FRIEDLANDER, M.L., TAYLOR, I.W., RUGG, A. & MUSGROVE, E.A. (1983). Method for analysis of cellular DNA content of paraffin-embedded pathological material using flow cytometry. *J. Histochem. Cytochem.*, **31**, 1333–1335.

HEDLEY, D.W. (1989). Flow cytometry using paraffin-embedded tissue: five years on. *Cytometry*, **10**, 229–241.

HOSAKA, Y., RAINWATER, L.M., GRANT, C.S., YOUNG, W.F., FARROW, G.M., VAN HEERDEN, J.A. & LIEBER, M.M. (1987). Adrenocortical carcinoma: nuclear deoxyribonucleic-acid ploidy studied by flow cytometry. *Surgery*, **102**, 1027–1034.

HOUGH, A.J., HOLLIFIELD, J.W., PAGE, D.L. & HARTMANN, W.H. (1979). Prognostic factors in adrenal cortical tumors. A mathematical analysis of clinical and morphological data. *Am. J. Clin. Pathol.*, **72**, 390–399.

JOENSUU, H., KLEMI, P. EEROLA, E. & TUOMINEN, J. (1988*a*). Influence of cellular DNA content on survival in differentiated thyroid cancer. *Cancer*, **58**, 2462–2467.

JOENSUU, L.L. & KLEMI, P.J. (1988*b*). DNA aneuploidy in adenomas of endocrine glands. *Am. J. Pathol.*, **132**, 145–151.

KANEKO, Y., KANDA, N., MASEKI, N., SUKARAI, M., TSUCHIDA, Y., TAKEDA, T., OKABE, I. & SUKARAI, M. (1987). Different karyotypic patterns in early and advanced stage neuroblastomas. *Cancer Res.*, **47**, 311–318.

LOOK, A.T., HAYES, F.A., NITSHKE, R., MCWILLIAMS, N.B. & GREEN, A.AQ. (1984). Cellular DNA content as a predictor of response to chemotherapy in infants with unresectable neuroblastoma. *N. Eng. J. Med.*, **311**, 231–235.

MATHEWS, D.E. & FAREWELL, V. (1985). *Using and Understanding Medical Statistics*, Karger: Basel.

MOOLENAAR, A.J., NIEWINT, J.W.M. & OEI, I.T. (1977). Estimation of o,p'-DDD in plasma by gas-liquid chromatography. *Clin. Chim. Acta*, **76**, 213–218.

OPPEDAL, B.R., STORM-MATHISEN, I., LIE, S.O. & BRANDTZAEG, P. (1988). Prognostic factors in neuroblastoma. *Cancer*, **62**, 772–780.

PADBERG, P.C., LAURITZEN, I., ACHILLES, E., HOLL, K., BRESSEL, M., KLOPPEL, G., DRALLE, H. & SCHRODER, S. (1991). DNA cytophotometry in adrenocortical tumours: a clinicomorphological study of 66 cases. *Virchows Archiv. A. Pathol. Anat.*, **419**, 167–170.

RODENBURG, C.J., CORNELISSE, C.J., HEINTZ, P.A.M., HERMANS, J. & FLEUREN, G.J. (1987). Tumour ploidy as a major prognostic factor in advanced ovarian cancer. *Cancer*, **59**, 317–323.

SAMAAN, N.A. & HICKEY, R.C. (1987). Adrenocortical carcinoma. *Sem. Oncol.*, **14**, 292–296.

SCHELFHOUT, L.J.D.M., CORNELISSE, C.J., GOSLINGS, B.M., HAMMING, J.F., KUIPERS-DIJKSHOORN, N.J., VAN DE VELDE, C.J.H. & FLEUREN, G.J. (1990). Frequency and degree of aneuploidy in malignant neoplasms. *Int. J. Cancer*, **45**, 16–20.

VAN SLOOTEN, H., MOOLENAAR, A.J., VAN SETERS, A.P. & SMEENK, D. (1984*a*). The treatment of adrenocortical carcinoma with o,p'-DDD: prognostic implications of serum level monitoring. *Eur. J. Clin. Oncol.*, **20**, 47–53.

VAN SLOOTEN, H. (1984*b*). *Het bijnierschorscarcinoom*. Thesis. pp. 90–102. Pasmans: 's Gravenhage, The Netherlands.

VAN SLOOTEN, H., SCHABERG, A., SMEENK, D. & MOOLENAAR, A.J. (1985). Morphological characteristics of benign and malignant adrenocortical tumors. *Cancer*, **55**, 766–773.

VINDELOW, L.L., CHRISTENSEN, I.J. & NISSEN, N.I. (1983). A detergent trypsin method for the preparation of nuclei for flow cytometric DNA analysis. *Cytometry*, **3**, 323–327.

WEISS, L.M. (1984). Comparative histologic study of 43 metastasizing and non-metastasizing adrenocortical tumors. *Am. J. Surg. Pathol.*, **8**, 163–169.